# In Vitro Analysis of the Anti-Inflammatory Effect of Inhomogeneous Static Magnetic Field-Exposure on Human Macrophages and Lymphocytes

**DOI:** 10.1371/journal.pone.0072374

**Published:** 2013-08-26

**Authors:** Cristian Vergallo, Luciana Dini, Zsuzsanna Szamosvölgyi, Bernardetta Anna Tenuzzo, Elisabetta Carata, Elisa Panzarini, János F. László

**Affiliations:** 1 Department of Biological and Environmental Science and Technology, University of Salento, Lecce, Italy; 2 National Institute of Pharmacy, Budapest, Hungary; 3 Department of Computer Science, University of Debrecen, Debrecen, Hungary; Universidad Pablo de Olavide, Centro Andaluz de Biología del Desarrollo-CSIC, Spain

## Abstract

The effect of inhomogeneous static magnetic field (SMF)-exposure on the production of different cytokines from human peripheral blood mononuclear cells (PMBC), *i.e.*, lymphocytes and macrophages, was tested *in vitro*. Some cultures were activated with lipopolysaccharide (LPS) at time point −3 h and were either left alone (positive control) or exposed to SMF continuously from 0 until 6, 18, or 24 h. The secretion of interleukin IL-6, IL-8, tumor necrosis factor TNF-α, and IL-10 was tested by ELISA. SMF-exposure caused visible morphological changes on macrophages as well as on lymphocytes, and also seemed to be toxic to lymphocytes ([36.58; 41.52]%, 0.308≤*p*≤0.444), but not to macrophages (<1.43%, *p*≥0.987). Analysis of concentrations showed a significantly reduced production of pro-inflammatory cytokines IL-6, IL-8, and TNF-α from macrophages compared to negative control ([56.78; 87.52]%, *p* = 0.031) and IL-6 compared to positive control ([45.15; 56.03]%, *p* = 0.035). The production of anti-inflammatory cytokine IL-10 from macrophages and from lymphocytes was enhanced compared to negative control, significantly from lymphocytes ([−183.62; −28.75]%, *p* = 0.042). The secretion of IL-6 from lymphocytes was significantly decreased compared to positive control ([−115.15; −26.84]%, *p* = 0.039). This massive *in vitro* evidence supports the hypotheses that SMF-exposure (i) is harmful to lymphocytes in itself, (ii) suppresses the release of pro-inflammatory cytokines IL-6, IL-8, and TNF-α, and (iii) assists the production of anti-inflammatory cytokine IL-10; thus providing a background mechanism of the earlier *in vivo* demonstrated anti-inflammatory effects of SMF-exposure.

## Introduction

Today, due to the preceding research work and its dissemination, it is widely accepted that static magnetic field (SMF)-exposure can achieve well defined observable responses from cells and living subjects under a broad range of experimental and clinical physiological and pathological conditions [1]–[5]. In contrast to the number of phenomenological observations and descriptions, little knowledge has accumulated about the background mechanisms of action [6], although the elective site of action of SMF seems to be the plasma membrane [7]–[9]. The probable reason for this is the lack of systematic research. Valuable experiments and clinical trials could not contribute to overall research as much as they would have deserved due to incomplete description of different magnetic inductions and their spatial gradients representing a 6D space, magnet arrangements, etc. Missing published data often hinder the replication of otherwise extremely important and valuable research projects.

The most frequent use of SMF-exposure is represented in MRI (Magnetic Resonance Imaging) devices nowadays. Although the MRI diagnosis requires 3 different types of magnetic fields, the *B*_0_ component is static. Research concerning the biological effects of SMF-exposure in humans diverges into two paths: the compilation of (i) epidemiological evidence that SMF-exposure does not imply health hazards and conversely, (ii) evidence of beneficial effects may raise SMF-exposure into an evidence-based medical therapy option. Reviews on the topic are available from WHO (World Health Organization) [10] and more recently from SCENIHR (Scientific Committee on Emerging and Newly Identified Health Risks) of the European Commission [11]. Russian scientists were specifically devoted to clarify the mechanisms behind magneto-therapy (meaning electromagnetic field irradiation); for a recent review see [12].

In particular, the production of pro-inflammatory cytokines interleukin IL-6 and tumor necrosis factor TNF-α, and that of the anti-inflammatory cytokine IL-10 was studied in the work of Salerno *et al.* [13] *in vitro* on human peripheral blood mononuclear cells (PBMC) under 0.5 T SMF continuous exposure for 24 h. The authors did not find any significant changes in the release of any cytokines. Aldinucci *et al.* [14] published similar conclusions of a similar study concerning pro-inflammatory cytokines IL-6 and TNF-α on identical subject cells as Salerno *et al.* [13], but the magnetic induction was continuous 4.75 T for 1 h. IL-8 was monitored by Sontag [15] in his *in vitro* study on human promyelocite cells (HL-60) exposed to 0–1.2 mT for a time period of 15 min. He neither found significant differences to unexposed control. Lin *et al.* [16] executed an *in vivo* experimental series on mice. A group of mice was exposed to SMF of 0.25 T for 2 h preceding a 50 mg/kg lipopolysaccharide (LPS) challenge. The authors did not find any significant change in the IL-6 and TNF-α secretion levels compared to positive control (LPS challenge only). However, Wang *et al.* [17] exposed human embryoid body derived cells to 0.23–0.28 T for 1–4 days continuously and found a significant increase in the release of IL-6.

If *in vitro* evidence was found on the SMF-exposure induced inhibition or assistance of the release of pro- and anti-inflammatory cytokines, a possible background mechanism of *in vivo* experienced antinociceptive effects in invertebrates [18], in mice [19]–[25], and in humans [26] would be supported. Therefore, the present set of *in vitro* experiments was designed to prove the null-hypotheses that exposure of PBMC to an inhomogeneous SMF for up to 24 h leads to a significant change in the:

1. production of IL-6, IL-8, TNF-α, and IL-10 compared to negative (unexposed) control;
2. LPS-activated production of IL-6, IL-8, TNF-α, and IL-10 compared to positive (LPS-activated, unexposed) control.

## Materials and Methods

### Ethics Statements

Human blood samples were obtained by buffy coats supplied by the Hospital S. Giuseppe da Copertino, Lecce, Italy. Donors were anonymous to us. The need of donor consent was waived by the Ethics Committee. The use of buffy coat was acknowledged by the Comitato Etico dell'ASL LE, Lecce, Italy (Ethics Committee of the Health Service of Lecce). This Ethics Committee is an independent organization that is working under the Declaration of Helsinki and following the rules of Good Clinical Practices according to international and national laws and to the guidelines of the Italian National Committee of Bioethics.

### SMF-exposure

SMF was generated with an exposure system described in details as generator #11 in [19]. Shortly, the device consisted of an upper and lower iron plate covered with 10×10 mm cylindrical neodymium-iron-boron (NdFeB) N50 grade magnets (*B_r_* = 1.47 T). The lateral periodicity of the SMF was 10 mm. The individual magnets on both plates were placed next to each other with alternating polarity. Magnets facing each other on the 2 plates were oriented with opposite polarity. The plates were fixed in a holder with 50 mm vertical separation between the upper and lower magnet arrays thus realizing an exposure chamber size 140×140×46 mm. Magnetic coupling applied between the matrices (the upper and lower magnet arrays were coupled through vertical ferromagnetic plates). Magnetic field mapping was performed separately from the *in vitro* experiments by means of a 5 V calibrated ratiometric linear Hall-effect sensor of 12.3 mV/T sensitivity (model UGN3503, Allegro MicroSystems, Worcester, MA, USA). The typical peak-to-peak magnetic induction values along the axis of a NdFeB magnet in the isocenter were 476.7±0.1, 12.0±0.1, and 2.8±0.1 mT, whereas the average lateral gradient values between 2 neighbouring local extremes were 47.7, 1.2, and 0.3 T/m at 3, 15, and 25 mm from the surfaces of plates, respectively. Horizontal components of the SMF in the exposure chamber and all components of Earth’s magnetic field were regarded as stray field. Control samples were exposed to the geomagnetic field, magnetic induction values of which were about 4 orders of magnitude lower than those at the target position in the exposure chamber.

### Cells, Chemicals

PBMC (monocytes and lymphocytes) were isolated from human buffy coats of non-smoker healthy male donors between 24 and 45 years of age by Ficoll gradient separation. Over 95% pure peripheral blood lymphocytes were separated from monocytes by overnight adherence to plastic. Cells were cultured in 25 cm^2^ flasks (Iwaki, Tokyo, Japan) at a cell density of 10^5^ cells/ml in RPMI-1640 medium (Cambrex BioScience, Verviers, Belgium) supplemented with 10% (v/v) heat-inactivated fetal calf serum (Cambrex), 2 mM L-glutamine (Cambrex), 100 IU/ml penicillin and streptomycin in a humidified atmosphere of 5% CO_2_ at 37°C. Some cultures of monocytes and lymphocytes were activated with 1 µg/ml LPS for 3 h first and then were exposed to SMF for 6, 18, or 24 h. Controls cells were cultured under the same culture conditions, but remained unexposed to SMF. All chemicals were of analytical grade and were provided by Sigma-Aldrich (St. Louis, MO, USA) unless otherwise indicated. Optical density (OD) values were read by spectrophotometer (Ultrospec 4000 UV/Visible Spectrophotometer, Pharmacia Biotech, Stockholm, Sweden) at 450 nm (OD_450_).

### Light Microscope Observations

An Eclipse TS100 (Nikon, Kawasaki, Kanagawa Prefecture, Japan) inverted microscope was used to investigate morphological cellular shape changes also at time points 0, 6, 18, and 24 h.

### Cytokine Assays

Production of different cytokines: IL-6 (monocytes and lymphocytes), IL-8 (monocytes), IL-10 (monocytes and lymphocytes), and TNF-α (monocytes) were determined by ELISA (R&D Systems, Minneapolis, MN, USA) from the supernatants at time points 0, 6, 18, and 24 h.

### Timeline

**Figure 1:**
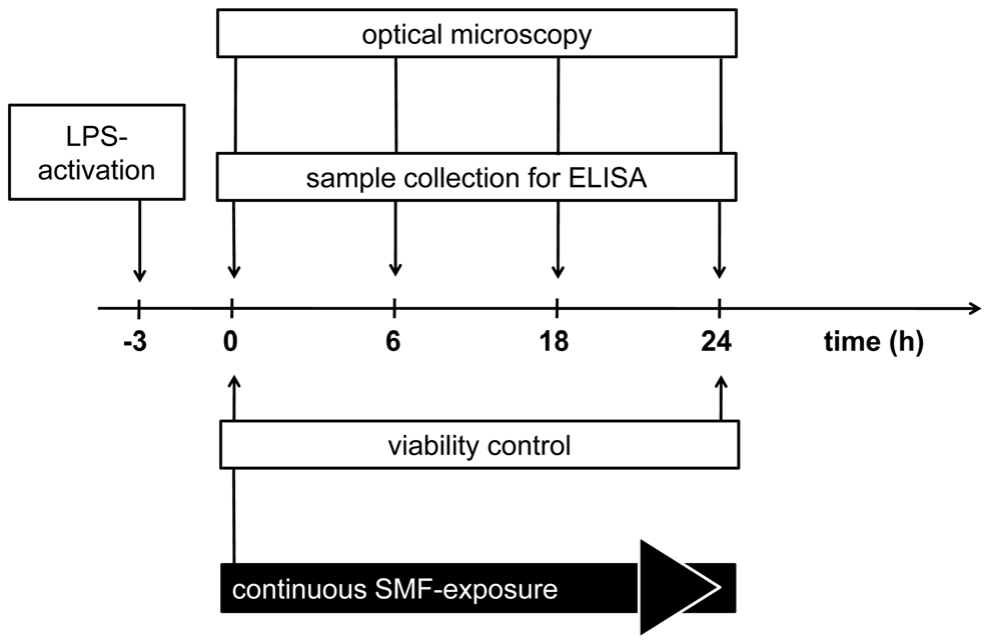
Timeline of the experiment. LPS stands for lipopolysaccharide, SMF for static magnetic field.

The timeline of the experiment can be seen in Fig. 1.

### Statistics

The primary outcome measure was the average OD_450_ value of a specific culture under a certain treatment at a given time point. Concentrations were derived from these values. First the best standard curve (*r* ^2^ = 99%) was set in accordance with the manufacturer’s instructions by plotting known concentrations of a certain cytokine expressed in pg/ml *vs* the relative OD_450_ read. Then we interpolated between known average OD_450_ values to get the desired concentration of the given cytokine.

Statistical measures were analyzed in a 2-sided manner in accordance with the null-hypotheses. Balanced, self-controlled group analysis of variances (2-way rANOVA) was applied for the estimate of the OD_450_ differences due to treatments. The treatment options and the time points were the factors. Treatment options were: none (negative control), LPS (positive control), SMF, or SMF+LPS. Time points were: 0, 6, 18, and 24 h. One-way ANOVA was applied in case of the concentrations. Games-Howell test was used for *post hoc* analysis of binary comparisons within time points (*n* = 9) and also in case of concentrations (*n* = 4 for negative and positive controls, *n* = 3 for SMF and SMF+LPS). A difference between group averages upon different treatments was accepted significant, if *p*<0.05 at the 95% confidence level. Probabilities below 0.001 are not shown numerically in the text.

Effect between treatment options *i* and *j* at a given *k* time point was defined as 
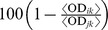
 in percent, where 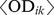 and 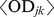 are averages of OD_450_ values at a given *k* time point, *n* = 9/treatment. *i* could only be SMF or SMF+LPS, the corresponding *j* could only be negative or positive (LPS) control, respectively. By definition the effect can be positive (inhibition) or negative (assistance). Concentrations (pg/ml), viabilities as well as effects (%) are presented in column graphs. Positive error bars in the figures denote standard deviation (SD). Identically scaled *y* axes were used for the presentation of effects allowing for immediate visual comparison.

## Results

**Figure 2:**
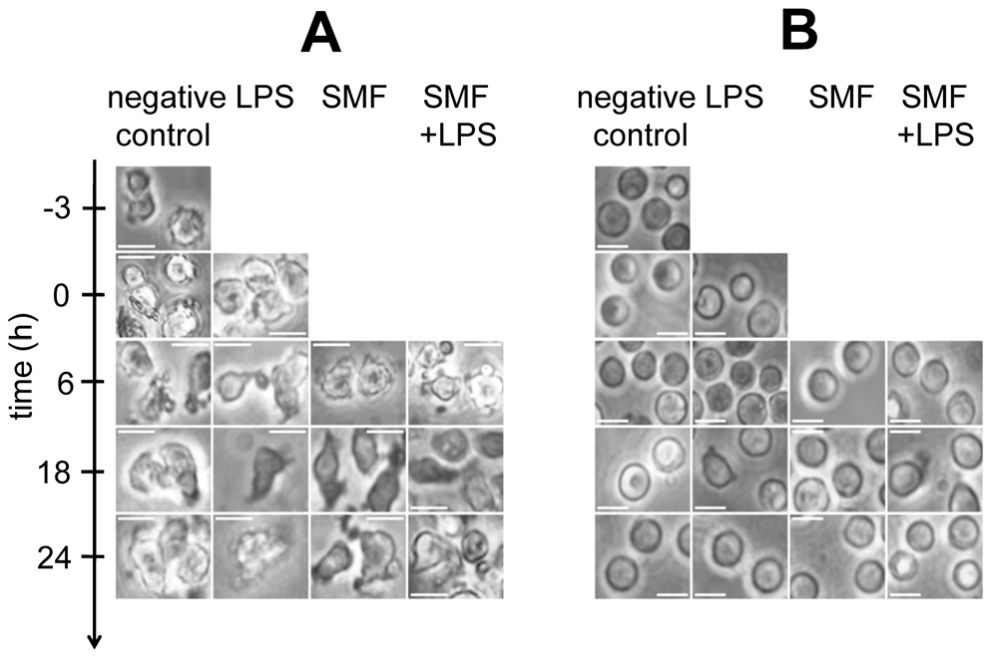
Evaluation of morphological changes. Light microscope images of the morphological changes of A) macrophages and B) lymphocytes. Negative control: no treatment; LPS: lipopolysaccharide-activation; SMF: static magnetic field-exposure; SMF+LPS: SMF-exposure combined with LPS-activation. Bar length = 10 µm. The time axis is out of scale.

Time-dependent cell shape modifications have been observed on SMF-exposed cells (on macrophages to a higher extent than on lymphocytes) all through the experiment. Fig. 2A and B show the morphological changes of macrophages and lymphocytes under different treatments negative control, positive control (LPS), SMF, and SMF+LPS. In particular, at time point 24 h macrophages showed an elongated shape while lymphocytes exhibited cell surface ruffling and indentations.

**Figure 3:**
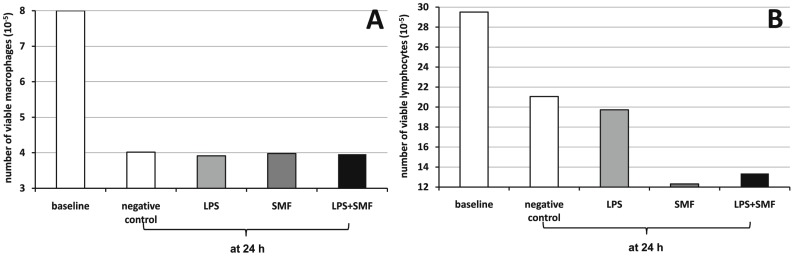
Evaluation of cell viability. Number of viable A) macrophages and B) lymphocytes at baseline and at 24 h. Cultures were exposed to different treatments: negative control: no treatment; LPS: lipopolysaccharide-activated; SMF: SMF-exposed; LPS+SMF: lipopolysaccharide-activated and SMF-exposed.

Viability of cells was controlled. Fig. 3A and B show the number of viable macrophages and lymphocytes, respectively at the baseline and at 24 h. Even if the spontaneous death of macrophages was about 49.81% during this period, none of the treatments seemed to have affected the procedure (remained between 50.31% and 51.09%). The maximum effect at 24 h between differently treated cultures to each other was 2.54% (LPS to negative control at 24 h). Lymphocytes showed higher sensitivity to the treatments: meanwhile apoptosis decreased the number of viable cells to 67.80% of the baseline value at 24 h, LPS caused a reduction of 92.50% only, but SMF alone 50.83%, and in combination with LPS 56.68% as compared to negative control at 24 h.

**Figure 4:**
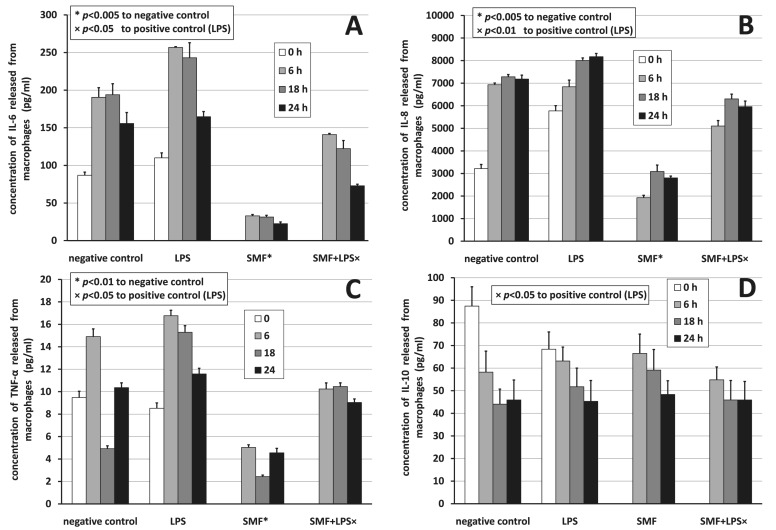
Cytokine profile release by macrophages. Concentration of A) IL-6, B) IL-8, C) TNF-α, and D) IL-10 released from macrophages in 24 h under different treatments. Positive error bars show standard deviation (SD) values. * and × denote significant differences (*p*<0.05) to corresponding negative or positive control (LPS), respectively as estimated with Games-Howell *post hoc* analysis.

Fig. 4A, B, C, and D show time-resolved concentration data for macrophages producing IL-6, IL-8, and TNF-α pro-inflammatory cytokines, and IL-10 anti-inflammatory cytokine, respectively. Different shades of the columns denote different time periods (0, 6, 18, and 24 h); treatment options are the independent variables. * and × mean significant differences *p*<0.05 compared to negative control and to positive control (LPS), respectively. For the 6–24 h time interval ANOVA provided *F*_crit_ = 4.07 for all cases analyzed and *F* = 27.92 (*p*<0.001), *F* = 3.13 (*p* = 0.087), *F* = 4.15 (*p* = 0.048), and *F* = 0.47 (*p* = 0.709) for IL-6, IL-8, TNF-α, and IL-10, respectively.

**Figure 5:**
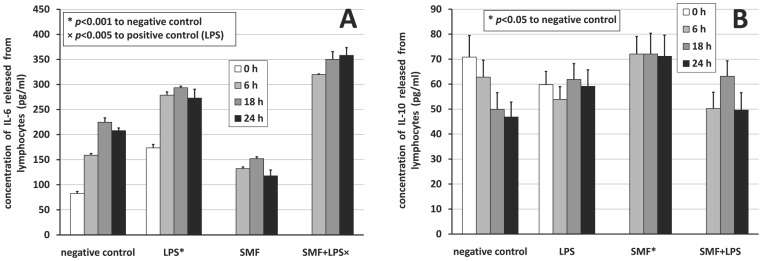
Cytokine profile release by lymphocytes. Concentration of A) IL-6 and B) IL-10 released from lymphocytes in 24 h under different treatments. Positive error bars show SD values. * and × denote significant differences (*p*<0.05) to corresponding negative or positive control (LPS), respectively as estimated with Games-Howell *post hoc* analysis.

Similarly to Fig. 4, Fig. 5A and B show the time-resolved concentrations for lymphocytes producing IL-6 and IL-10, respectively. Different shades and marks (* and ×) have the same meaning as in Fig. 4. ANOVA resulted in *F* = 4.88 (*p* = 0.033) and *F* = 3.01 (*p* = 0.095) for IL-6 and IL-10, respectively.

SMF-exposure was more efficient on macrophages than on lymphocytes, since macrophages secreted more cytokines than lymphocytes. Without taking the time-dependence into account at baseline, when no SMF treatment was yet used, but LPS had been given for 3 h already, the secretion of IL-6 and IL-8 from macrophages and IL-6 from lymphocytes started. LPS-activation hardly induced TNF-α production in the first 3 h. LPS as an activator seemed to work well in the model for macrophages as well as for lymphocytes mimicking the inflammatory situation *in vitro*: it enhanced the secretion of IL-6 significantly from lymphocytes (−50.61%, *p*<0.001 estimated by rANOVA), and the secretion of IL-6 (−23.33%, *p*<0.001), IL-8 (−17.04%, *p*<0.001), and TNF-α as well (−23.09%, *p*<0.001) from macrophages. LPS also induced a tendency to increase IL-10 release beyond the first 6 h. LPS-activated cells in the absence of SMF secreted the highest amounts of IL-8 at all times compared to negative control.

**Figure 6:**
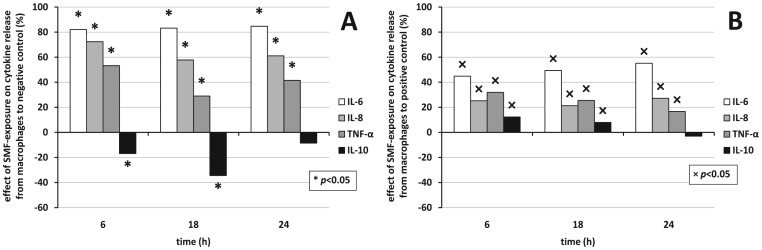
Effect of SMF exposure on cytokine release by macrophages. Effect of cytokine release from macrophages under SMF-exposure compared to A) negative and B) positive (LPS) control, respectively as estimated from OD_450_ data. * and × denote significant differences (*p*<0.05) to negative and positive control, respectively. Positive effect means inhibition, negative means assistance. Probabilities of significance can be found in Table 1.

**Figure 7:**
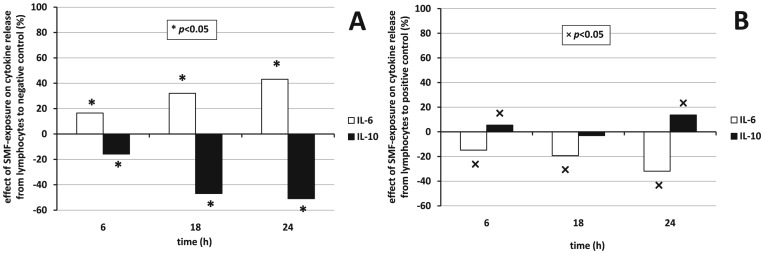
Effect of SMF exposure on cytokine release by lymphocytes. Effect of cytokine release from lymphocytes under SMF-exposure compared to A) negative and B) positive (LPS) control, respectively as estimated from OD_450_ data. * and × denote significant differences (*p*<0.05) to negative and positive control, respectively. Positive effect means inhibition, negative means assistance. Probabilities of significance can be found in Table 1.

The effect of cytokine release due to SMF-exposure was based on raw OD_450_ data, the time-dependent viability of cells was not considered. Positive or negative effect means if a treatment decreases or increases the OD_450_ values compared to respective control. All figures show a *y* axis with identical scale for easy visual comparison. The effect of SMF acting on cytokine secretion from macrophages compared to negative and positive control can be seen in Fig. 6A and B. The numerical results of probabilities of significance based on OD_450_ values are collected in Table 1. Similarly to Fig. 6A and B, the effect of SMF on cytokine release from lymphocytes compared to negative and positive control can be seen in Fig. 7A and B, respectively.

**Table 1:** Effect sizes and corresponding significances for OD_450_ and for concentration values of pro-inflammatory (IL-6, IL-8, TNF-α) and anti-inflammatory (IL-10) cytokines produced by macrophages or lymphocytes under lipopolysaccharide *(*LPS) activation and/or static magnetic field (SMF)-exposure in the 6–24 h time period.

		effect of SMF-exposure in OD_450_											
sourcecytokinetreatment to control		macrophages								lymphocytes			
		IL-6		IL-8		TNF-α		IL-10		IL-6		IL-10	
		SMF to negative control	SMF+LPS to positivecontrol	SMF to negativecontrol	SMF+LPS to positivecontrol	SMF to negative control	SMF+LPS to positivecontrol	SMF to negative control	SMF+LPS to positivecontrol	SMF to negative control	SMF+LPS to positive control	SMF to negative control	SMF+LPS to positive control
**exposure time (h) and ** ***p*** ** value or range**	**6**	82.06	44.84	72.33	25.2	53.25	31.91	−16.92	12.27	16.57	−14.82	−15.77	5.47
		*<0.001	×<0.001	*<0.001	×<0.001	*<0.001	×<0.001	*0.002	×<0.001	*<0.001	×<0.001	*<0.001	×0.021
	**18**	83.16	49.38	57.77	21.3	28.96	25.43	−34.46	7.89	32.08	−19.24	−46.84	−3.05
		*<0.001	×<0.001	*<0.001	×<0.001	*<0.001	×<0.001	*<0.001	×0.012	*<0.001	×<0.001	*<0.001	
	**24**	84.65	55.16	61.07	27.16	41.51	16.62	−8.58	−3.02	43.16	−31.91	−50.88	13.72
		*<0.001	×<0.001	*<0.001	×<0.001	*<0.001	×<0.001			*<0.001	×<0.001	*<0.001	×<0.001
		**effect of SMF-exposure in concentrations**											
**source** **cytokine** **treatment to control**		**macrophages**								**lymphocytes**			
		**IL-6**		**IL-8**		**TNF-α**		**IL-10**		**IL-6**		**IL-10**	
		**SMF to negative** **control**	**SMF+LPS to positive** **control**	**SMF to negative** **control**	**SMF+LPS to positive** **control**	**SMF to negative** **control**	**SMF+LPS to positive** **control**	**SMF**	**SMF+LPS to positive** **control**	**SMF to negative** **control**	**SMF+LPS to positive** **control**	**SMF to negative** **control**	**SMF+LPS to positive** **control**
**exposure time (h)**	**6**	82.48	45.15	72.17	25.61	87.52	49.82	−13.88	12.82	4.91	−26.84	−28.75	−2.69
	**18**	83.33	49.95	57.35	21.95	69.75	35.47	−33.42	11.55	−3.83	−63.17	−111.18	−37.03
	**24**	84.76	56.03	60.52	28.17	56.78	23.91	−6.45	0.15	−12.20	−115.15	−183.62	−35.99
**significance ** ***p*** ** range**		*<0.005	×<0.05	*<0.005	×<0.01	*<0.01	×<0.05		×<0.05	*<0.001	×<0.005	*<0.05	

* and × denote significant differences (*p*<0.05) to negative and positive control, respectively as estimated with Games-Howell *post hoc* test.

SMF-exposure alone induced an anti-inflammatory response of macrophages and lymphocytes by strongly and significantly inhibiting the secretion of pro-inflammatory cytokines IL-6, IL-8, and TNF-α as compared to negative control. The effect of SMF-exposure on IL-10 anti-inflammatory cytokine presented an opposite trend; a moderate, but significant assistance could be seen.

LPS-activated cells in the presence of SMF strongly and significantly inhibited the secretion of pro-inflammatory cytokine IL-6, IL-8, and TNF-α from macrophages. These effects were significant. A surprising negative and with time monotonously decreasing effect occurred in case of IL-6 release from lymphocytes. The production of anti-inflammatory cytokine IL-10 underwent a slight, hardly significant inhibition as compared to positive control from either macrophages, or lymphocytes. The time dependence of the effect of cytokine secretion manifested itself in a monotonous rise in the inhibition in case of IL-6 for both sources compared to either control, but for lymphocytes compared to positive control. IL-8 production hardly varied with time following 6 h. TNF-αinhibition basically decreased with time, while IL-10 was the only examined cytokine the production of which correlated with the source, the absolute value of the assisting effect decreased in time for macrophages and increased for lymphocytes compared to negative control. LPS-activation in the presence of SMF-exposure had a mild effect (below 20%) on the release of IL-10 for either source. Meanwhile, SMF-exposure from macrophages showed a trend of diminishing action of the release of cytokines with the time of exposure, the action from lymphocytes seemed to be gradually enforced.

Statistical analysis (see Table 1 for a comprehensive view) of the concentrations showed that null-hypothesis 1 (*i*.*e*., effect of SMF-exposure to negative control) held for IL-6, IL-8, and TNF-α for 6–24 h with a significant inhibition between 56.78% and 87.52% for the release of cytokines from macrophages hindering the production of pro-inflammatory cytokines. SMF-exposure basically remained ineffective on the IL-6 release from lymphocytes (<|12.20%|). SMF-exposure assisted the release of anti-inflammatory cytokine IL-10 from macrophages as well as from lymphocytes (between −183.62% and −6.45%) significantly for lymphocytes, insignificantly for macrophages. Null-hypothesis 2 (*i*.*e*., effect of SMF-exposure to positive control) also held for all pro-inflammatory cytokines. Pro-inflammatory cytokine release was inhibited from macrophages (effect of 0.15%–56.03%) by the SMF-exposure (significantly for IL-6), while exposure surprisingly significantly assisted the release of IL-6 from lymphocytes (between −115.15% and −26.84%). Anti-inflammatory cytokine IL-10 release from macrophages was mildly and insignificantly inhibited from macrophages and assisted from lymphocytes.

## Discussion

Normally unstimulated human primary PBMC undergo spontaneous cell death in culture. This is the reason why the decrease in the viability of macrophages as well as lymphocytes in the cultures together with the withdrawal of the release of pro-inflammatory cytokines IL-6, IL-8, TNF-α, and the increase of anti-inflammatory cytokine IL-10 was happening simultaneously in the present experiment. In previous studies it was already demonstrated that 6 mT SMF-exposure applied for up 24 h to human U937 promonocytic cells [1] or to freshly isolated human lymphocytes [3] did not significantly exert any toxic or apoptogenic effect. However, in agreement with the data reported here (Fig. 2), 6 mT SMF-exposure affected cell shape [7] as a result of cytoskeleton rearrangements [1] or by influencing the structural components of plasma membrane directly [9].

In the present study, the effects observed on macrophages can be directly compared without weighting with the degree of lost viability, since the number of macrophages hardly depended on the treatment options (including no treatment) in the first 24 h period.

It is certainly interesting in itself what a strong adverse effect SMF-exposure exerted on the viability of lymphocytes. Although previous *in vitro* studies suggested otherwise for extremely weak SMF on human lymphocytes [3], the authors attribute the effect to the magnetic induction at the target site in the present experimental series. Namely, magnetic induction exceeded 6 mT by almost 2 orders of magnitude at average peak-to-peak value. On the other hand, the elevated level of cell death is in harmony with the results presented by Lee *et al.* [27], who found severe DNA breaks due to exposure with a combined magnetic field of a 3 T clinical MRI running different scanning protocols for durations between 22 and 89 min *in vitro* on human lymphocytes.

There is an opposition in the dependence of the SMF-exposure induced cytokine releasing action on macrophages and lymphocytes as a function of exposure duration. Lymphocytes are fully differentiated cells, while macrophages – in the absence of specific inducers – are not fully differentiated. Thus, lymphocytes and (monocyte-derived) macrophages have different genetic expression. This is in accordance with the different roles of the cells: macrophages may produce cytokines immediately upon stress; the production of cytokines by lymphocytes however, is of secondary importance for the lymphocytes. As against macrophages, lymphocytes use receptors to bind antigens, and the role of SMF-exposure on some receptors has already been shown [21], [28]. Therefore, we might hypothesize that the response evoked by strong SMF-exposure could be different for lymphocytes and macrophages as already demonstrated by Tenuzzo *et al.* [2] for 6 mT SMF-exposure.

Gyires *et al.* [21] published a classical pharmacological analysis of the action of SMF-exposure in a mouse model of acute visceral pain with the same SMF generators as used in the present study. They discovered the significant role of opioid receptors, especially that of μ-opioid receptors, and also succeeded in excluding the contribution of κ-opioid receptors. The authors speculated that the strong analgesic effect of SMF-exposure may have been due to the release of either/both β-endorphin or/and endomorphin-2 in the spinal cord. Later László and Hernádi [18] verified in *Helix pomatia*, in a land snail model that the thermal nociceptive threshold depended on the preceding 20–40 min long SMF-exposure to either homogeneous SMF of 147 mT or to an inhomogeneous SMF-exposure, where SMF was identical to that present. With the application of naloxone pretreatment they could reveal the mediatory role of opioid receptors in the SMF-induced antinociception. There is also evidence collected that low-dose naloxone with morphine has a similar effect of pro- and anti-inflammatory cytokines as presented here [29]. This suggests that the action of SMF-exposure may be similar to that of serum morphine in the sense that naloxone can moderate both, and the secretion of pro- and anti-inflammatory cytokines was also observed.

Sándor *et al.* [20] used the same SMF generator to prove that the capsaicin-sensitive fibers also mediated the anti-hyperalgesic action of SMF-exposure in a mouse acute peripheral pain model. Carrageenan, TNF-α, IL-6, and IL-8 were reported to induce hyperalgesia [30]. The downregulation of the pro-inflammatory cytokines by the SMF-exposure may explain why Sándor *et al.* [20] noticed the significant decrease of hyperalgesia induced by carrageenan in their peripheral pain model. These observations can now be linked to the altered cytokine release characteristics of macrophages and lymphocytes from PBMC.

The upregulation of IL-6 and TNF-α expression in case of chronic constriction injury has been shown recently [31]. If SMF-exposure acts against chronic constriction injury, the extraordinary beneficial effect reported by Antal and László [23] in their *in vivo* experiment of a chronic neuropathy mouse model can – at least partially – be understood. Namely, they used nerve ligature to achieve neuropathic pain.

SMF-exposure was also shown to significantly prolong the time of LPS-induced premature birth occurrence [32]. A background mechanism for this beneficial effect can be the moderation of the inflammatory response as SMF-exposure acts on the pro-inflammatory cytokine release.

The present results are in fair harmony with those of Wang *et al.* [17], who applied 0.23–0.28 T exposure on human embryoid body derived cells continuously for 1–4 days. They experienced a short-term (<24 h) activation of IL-6 involved the coordinate up-regulation of toll-like receptor-4 (TLR4) with complementary changes to NEU3 and ST3GAL5 that reduced ganglioside GM3 in a manner that augmented the activation of TLR4 and IL-6. The authors suggested a plausible mechanism for the attenuation of cellular responses to SMF by the loss of GM3. They also observed SMF-mediated morphological changes and biochemical markers indicative of pre-oligodendrocyte differentiation at the cellular level.

In conclusion, the present *in vitro* experiments demonstrate convincing evidence that exposure to a strong, inhomogeneous SMF for up to 24 h has a significant inhibitory effect on the release of pro-inflammatory cytokines IL-6, IL-8, and TNF-α from macrophages as compared to negative, untreated control. The assistance SMF-exposure exerts on the production of anti-inflammatory cytokine IL-10 from lymphocytes was also found significant. LPS-activation reduces the differences between treated and untreated cultures, but IL-6 release from macrophages remains significantly inhibited upon SMF-exposure. The SMF-exposure assisted release of IL-6 from lymphocytes remains a challenging effect to be dealt with in the future. The present experiments may be regarded as proof that SMF-exposure in fact has a beneficial effect on human macrophages and lymphocytes *in vitro*, and as such SMF-exposure should be a worthy candidate of further investigations in clinical trials including diseases with inflammatory background.
